# Book Reviews

**Published:** 1907-01

**Authors:** 


### BOOK REVIEWS.

*Golden Rules of Pedratrics.* Aphorisms, observations and precepts on the science and art of Pedratrics: Giving the Practical Rules for Diagnosis and Prognosis; the Essentials of Infant Feeding, and the Principles of Scientific Treatment. By John Zahorsky, A. B., M. D., Clinical Prof. of Pedratrics, Washington University Medical Department, St. Louis, etc., etc. With introduction by E. W. Saunders, M. D. The C. V. Mosby Medical Book Co., St. Louis, Mo., pp. 362. $3.00 net.

Dr. Zahorsky has collected many interesting and valuable facts which he calls "Golden Rules." These are arranged in groups according to the different subjects to which they are related, concluding the work is a Therapeutic Addendum and Formulary containing many prescriptions suitable for the various diseases of children.

---

*The Practitioners Visiting List* for 1907. An invaluable pocket-sized book containing memoranda and data important for every physician, and ruled blanks for recording every detail of practice. The Weekly, Monthly and 30-Patient Perpetual contain 32 pages of data and 160 pages of classified blanks. The 60-Patent Perpetual consists of 256 pages of blanks alone. Each in one wallet-shaped book, bound in flexible leather, with flap and pocket, pencil and rubber, and calendar for two years. Price by mail, postpaid, to any address, $1.25. Thumb-letter index, 25 cents extra. Descriptive circular showing the several styles sent on request. Lea Brothers & Co., Publishers, Philadelphia and New York, 1906.

Being in its twenty-first year of issue, *The Practitioners' Visiting List* embodies the results of long experience and study devoted to its development and perfection.

It is issued in four styles to meet the requirements of every practitioner: "Weekly," dated for 30 patients; "Monthly," undated for 120 patients per month; "Perpetual," undated, for 30 patients weekly per year, and "60 Patients," undated, for 60 patients weekly per year.

*Stenhouse and Ferguson's Epitome of Pathology.* By John Stenhouse, M.D., of the University of Toronto, and John Ferguson, M. D., Toronto, Canada. 12mo., 285 pages, amply illustrated. Cloth, $1.00 net. Lea Brothers & Co., Publishers, Philadelphia and New York, 1906. (Lea's Series of Medical Epitomes. Edited by Victor C. Pedersen, M.D.)

The volume on *Pathology* brings near to completion the *Medical Epitome Series,* which is to comprise twenty-three manuals authoritatively covering the whole field of medical education. Twenty of these are now ready, leaving only the volumes on *Nose* and *Throat, Gynecology, and Hygiene* to be published shortly. Drs. Stenhouse and Ferguson devote the first half of their work to *General Pathology,* after which the *Special Pathology* of the various organs and systems is considered. This arrangement conforms to the modern method of handling the subject, so that this excellent epitomization will serve not only the student in acquiring a well assorted knowledge, but also the practitioner who desires to post up on the leading points. Mastery of the information so easily presented in this compact volume will qualify its readers on the essentials of the subject and facilitate the work of those who desire to pursue it further in the larger treatises.

---

*A Dictionary of Medical Science,* containing a full explanation of the subjects and terms connected with all its various branches, by Robley Dunglison, A. M., LL.D., late Professor of Institutes of Medicine and Medical Jurisprudence in the Jefferson Medical College of Philadelphia, etc. Twenty-third edition thoroughly revised and re-edited by Thomas L. Stedman, M. D. Octavo volume 1212 pages, with 577 illustrations, etc. Cloth $8.00 net; leather $9.00 net; half morocco $9.50 net. Lea Bros. and Co., New York and Philadelphia.

More than seventy-six years ago, Dr. Robley Dunglison gave to the English speaking world a medical dictionary so excellent that twenty-two editions have been called for. The large number of new words added in each revision may be taken as an accurate index to the growth of the various departments of medicine and its allied sciences. Especially valuable is increase of words bearing on Bacteriology, Materia Medica, Therapeutics and Synthetic Chemistry.

No physician can keep abreast with his colleagues unless he is in touch with the new words which represent the advances and improvements so necessary for his success and scientific work.

There is no dictionary in the English language held in higher esteem, and justly so, than Dunglison's. It is complete, practical and up-to-date.

---

*Grayson's Laryngology.* The Diseases of the Nose, Throat and Ear. By Charles P. Grayson, M. D., Clinical Professor of Laryngology, Medical Department, University of Pennsylvania. New (2d) edition, revised and enlarged. Octavo, about 550 pages, with 152 engravings and 15 plates in black and colors. Cloth, $4.00, net. Lea Brothers & Co., Philadelphia and New York, 1906.

The distinguishing feature of Dr. Grayson's treatise on the Nose, Throat and Ear, in its first edition was the manifest skill with which he selected exactly what his readers would desire to know, and the exceeding clarity of his presentation. A mind which arranges knowledge in the order of its importance, and holds it clearly in view, is incapable of verbosity, and accordingly, the author has found space for the whole of a major specialty in a very convenient volume. This he has achieved while allotting exceptionally full consideration to the main objective, treatment. He has given constant thought to those who wish to know not only *what* to do, but also *how* to do it. He has been guided by his experience in selecting those measures which have been most often successful in subduing the symptoms of a disease and shortening its duration. The plan of a work so conceived is scarcely susceptible of improvement, hence the new edition adheres closely to it, embodying of course a thorough revision to the latest date, with much new matter and many new illustrations. It is a volume which appeals equally to the interest of students, practitioners and specialists.

